# The pH of Drinking Water and Its Effect on the pH of Urine

**DOI:** 10.7759/cureus.47437

**Published:** 2023-10-21

**Authors:** İlker Yıldırım, Hüseyin Koçan

**Affiliations:** 1 Anesthesiology and Reanimation, Tekirdağ Namık Kemal University, Faculty of Medicine, Tekirdağ, TUR; 2 Urology, Health Sciences University, Kanuni Sultan Süleyman Training and Research Hospital, İstanbul, TUR

**Keywords:** tissue fluid, kidney function, urine ph, water ph, wistar albino rat

## Abstract

Aim: The aim was to determine whether urine pH changed or not with different pH values of drinking water. With the results obtained from animal studies, comments can be made about the effect of water with different pH levels that people drink on kidney stones.

Method: A total of 24 Wistar Albino rats were divided into three groups containing eight rats each: the first group was given water with pH 5.5, the second group was given water with pH 7 and the third group was given water with pH 8.2 in the same environment and conditions during 13 days. All rats consumed water in line with their natural feeding habits. All rats had urine pH measurements performed and recorded every day at the same time. The groups were later compared in terms of daily pH values.

Results: When daily urine pH values were compared, there were statistically significant differences between pH measurements on the first, fourth and seventh day (p=0.02, p=0.017 and p=0.007, respectively). When first-day values are compared with post-hoc analyses, the urine pH in Group 2 was identified to be lower compared to Group 1 and Group 3 (p<0.001). When the fourth-day values were assessed, the urine pH of Group 2 was observed to be higher than Group 1 and Group 3 (p<0.001). On the seventh day, Group 3 had higher urine pH compared to the other groups (p<0.001).

Conclusion: The variation in drinking water pH does not directly change urine pH; however, it causes a change in the urine pH on different days.

## Introduction

Normal urine pH values are between pH 4.6 and pH 8.0. High pH in urine may occur in cases such as kidney failure, impaired urine concentration ability, urinary tract infections, vomiting and acidosis due to renal tubes. In addition, blood transfusions and excessive intake of alkaline-forming substances such as baking soda are among the effective causes. Low pH value in the urine can be caused by taking acidic drugs, acidosis, diabetes mellitus, chronic nephritis, gout, leukemia, high protein diet, taking saccharin, vitamin C deficiency and it may occur due to reasons such as acute joint rheumatism [1]. The minerals contained in foods make them acidic or alkaline. While iodine, chlorine, sulfur and phosphorus have an acidic effect, magnesium, potassium, calcium, sodium, iron and manganese have an alkaline effect [2,3]. Urine pH may provide benefits in managing dysuria, especially in urinary system stone disease and for some symptoms. The prevalence of urolithiasis varies by race and gender, and the incidence varies with age [4,5]. The recurrence of stone disease makes it an important health problem that causes a financial burden on country economies and may affect the quality of life of patients [6]. Studies show that the recurrence of stone disease reaches up to 50% within 10 years [7,8]. As a result, preventing stone disease is among the important health problems. The most important approaches are the prevention of kidney stones with diet and lifestyle changes [9,10]. There are different factors affecting the content of stones and one of these is urine pH. Crystallization of solutions in urine is directly related to urine pH. The solubility of uric acid increases as urine pH increases, while cysteine crystal formation increases in normal urine pH, struvite stones form in alkali urine pH and urine pH may affect the formation of stones related to medication [11]. In spite of some clinicians believing that alkalizing urine will provide benefit by reducing some symptoms like dysuria and organizing treatment accordingly, limited studies have not identified a difference in urine pH values between disease and disease-free periods in patients with these symptoms [12]. Bottled and tap waters display much variability in pH values from alkali to acid. Based on this difference, we aimed to determine whether urine pH changes or not with the pH of drinking water.

## Materials and methods

Experiment

The study included 24 Wistar Albino rats with a median weight of 248 g (240-260 g). These animals were housed according to the recommendations in the care and use of laboratory animals guidelines. The study was planned in this way due to the similarity of the physiology of Wistar Albino rats to human physiology and the controllability of external factors that can change the urine pH in their diet [13]. The experiment was completed after receiving permission from the animal experiments ethics committee of İstanbul Mehmet Akif Education and Research Hospital and was completed in the same location. Rats were divided into three groups of eight rats each: the first group was given drinking water with pH 5.5, the second group was given drinking water with pH 7 and the third group was given drinking water with pH 8.2. All rats were fed in the same environment and conditions for 13 days. All rats consumed water in line with their natural feeding habits. All rats had urine pH measurements taken at the same time every day and results were recorded.

The acidity and base values of fluids are expressed as values from 0 to 14. While pH 7 is neutral, the values higher than 7 are alkaline (basic), while the values lower than 7 are acidic. In a temperate climate, the US National Academies of Sciences, Engineering and Medicine recommend 3.7 liters of daily fluid intake for healthy adult men and 2.7 liters for women. Another study recommended drinking more than 2 L of water daily to reduce stone recurrence [14,15]. The US Environmental Protection Agency and World Health Organization (WHO) recommend pH be in the interval from 6.5 to 9 for drinking water quality [16]. The WHO did not find it necessary to recommend any pH value for health [16]. In fluids, pH can be measured with a pH meter or color comparator method. The color comparator involves adding an indicator reactive that colors water to the sample. The color intensity is proportional to the pH of the sample. This color is then matched to a standard color scheme to determine the pH [17]. In our study, the color comparator method was used for pH measurements. The US Food and Drug Administration defines bottled water sold for human consumption as displaying much variability linked to the source and brand [18].

Statistical analysis

Normality was examined with the Shapiro-Wilk test, histogram, Q-Q plot and box plot graphs. Data are given as median, minimum and maximum. We used G-power analysis to recruit subjects for our study. It was determined that there should be a minimum of eight subjects for each group. Groups were compared using the Kruskal-Wallis one-way analysis of variance. Multiple comparisons were performed with the Dunn test. The limit of significance was p<0.05 and double-sided. Analyses were performed using the NCSS 10 (2015; Kaysville, UT, USA) software program.

## Results

The daily monitoring of statistical data for the groups was given in Table 1. There were differences between the groups on the first day, fourth day and seventh day (p=0.02, p=0.017 and p=0.007, respectively). There were no differences on the other days (Table 2). On the fourth day, there were differences between the pH 5.5 and pH 7 groups (p<0.01) and pH 7 and pH 8.2 groups (p<0.05). The values for the pH 7 group were higher than for pH 5.5 and pH 8.2 groups (pH 5.5: 7 (6-7), pH 7: 8 (7-9); pH 8.2: 7 (6-9)). On the seventh day, there were differences between the pH 5.5. and pH 8.2 groups (p<0.01) and between the pH 7 and pH 8.2 groups (p<0.05). The value for pH 8.2 group was higher than for pH 5.5 and pH 7 groups (pH 5.5: 7 (6-7), pH 7: 7 (6-8); pH 8.2: 8 (7-9)) (Table 3). It was confirmed that the data showed normal distribution with the Shapiro-Wilk test.

**Table 1:** Statistical Data for Daily Monitoring of Groups

Group			Days												
			1	2	3	4	5	6	7	8	9	10	11	12	13
pH 5.5	N	Valid	8	8	8	8	8	8	8	8	8	8	8	8	8
		Missing	0	0	0	0	0	0	0	0	0	0	0	0	0
	Mean		8.5	7.38	7.25	6.75	6.88	6.88	6.75	6.88	7.13	7	6.75	6.63	7.13
	Std. Deviation		0.756	1.302	1.282	0.463	0.354	0.354	0.463	0.835	0.991	0.926	0.886	0.518	0.354
	Minimum		7	6	6	6	6	6	6	6	6	6	6	6	7
	Maximum		9	9	9	7	7	7	7	8	8	8	8	7	8
	Percentiles	25	8	6	6	6.25	7	7	6.25	6	6	6	6	6	7
		Median	9	7.5	7	7	7	7	7	7	7.5	7	6.5	7	7
		75	9	8.75	8.75	7	7	7	7	7.75	8	8	7.75	7	7
pH 7	N	Valid	8	8	8	8	8	8	8	8	8	8	8	8	8
		Missing	0	0	0	0	0	0	0	0	0	0	0	0	0
	Mean		7.25	7.13	8.5	7.88	7.75	6.5	6.88	7.63	7	7.38	7.25	6.75	6.88
	Std. Deviation		1.035	0.991	0.535	0.641	0.886	0.535	0.835	0.518	0.926	0.518	0.707	0.707	0.641
	Minimum		6	6	8	7	7	6	6	7	6	7	6	6	6
	Maximum		9	9	9	9	9	7	8	8	8	8	8	8	8
	Percentiles	25	6.25	6.25	8	7.25	7	6	6	7	6	7	7	6	6.25
		Median	7	7	8.5	8	7.5	6.5	7	8	7	7	7	7	7
		75	8	7.75	9	8	8.75	7	7.75	8	8	8	8	7	7
pH 8.2	N	Valid	8	8	8	8	8	8	8	8	8	8	8	8	8
		Missing	0	0	0	0	0	0	0	0	0	0	0	0	0
	Mean		8.5	8.25	7.88	7	7.25	6.88	8.13	7.25	7.25	7.13	7.63	7	7.38
	Std. Deviation		0.535	0.886	0.835	1.069	0.707	1.126	0.835	0.707	0.707	0.835	1.061	0.756	0.518
	Minimum		8	7	7	6	6	6	7	6	6	6	6	6	7
	Maximum		9	9	9	9	8	9	9	8	8	8	9	8	8
	Percentiles	25	8	7.25	7	6	7	6	7.25	7	7	6.25	7	6.25	7
		50	8.5	8.5	8	7	7	6.5	8	7	7	7	7.5	7	7
		75	9	9	8.75	7.75	8	7.75	9	8	8	8	8.75	7.75	8

**Table 2:** Comparison of Kruskal-Wallis with one-way ANOVA for groups a. Kruskal Wallis Test b. Grouping Variable: Group

Test Statistics^a,b^			
Days	Chi-Square	df	Asymp. Sig.
1st day	7.83	2	0.02
2nd day	4.304	2	0.116
3rd day	5.042	2	0.08
4th day	8.119	2	0.017
5th day	5.166	2	0.076
6th day	1.729	2	0.421
7th day	9.851	2	0.007
8th day	3.82	2	0.148
9th day	0.283	2	0.868
10th day	0.757	2	0.685
11th day	3.19	2	0.203
12th day	1.15	2	0.563
13th day	3.309	2	0.191

**Table 3:** Multiple comparisons of groups Regular Test: Medians significantly different if z-value >1.9600 Bonferroni Test: Medians significantly different if z-value >2.3940

Kruskal-Wallis Multiple-Comparison Z-Value Test (Dunn's Test)				
Days	pH	pH 5.5	pH 7	pH 8.2
1st day	pH 5	<0.001	2.4777	0.1126
	pH 7	2.4777	<0.001	2.3651
	pH 8	0.1126	2.3651	<0.001
4th day	pH 5	<0.001	2.6759	0.4900
	pH 7	2.6759	<0.001	2.1860
	pH 8	0.4900	2.1860	<0.001
7th day	pH 5	<0.001	0.3006	2.8559
	pH 7	0.3006	<0.001	2.5553
	pH 8	2.8590	2.5553	<0.001

## Discussion

We could not find any previous study investigating the relationship between water pH and urine pH. That's why our work is important. The pH of bottled waters is mainly in the 5 to 8.6 interval. In our study, the bottled waters with the lowest and highest pH values accessible in our region were used. The drinking water pH was 5.5 for the first group, pH 7 for the second group, and 8.2 for the third group. The pH of drinking water does not cause a regular change in urine pH; However, it causes changes to urine pH on different days. It can be seen from literature screening that the potential benefits and harms of water pH is a topic that has not been studied much by researchers. One study stated that the use of neutral and/or alkali water may prevent degradation of dental structure and demineralization in people with dry mouths, and they warned health professionals about the need to pay attention to this topic [19,20]. One of these studies showed that people eating acid-content or alkaline-content diets changed the urine pH in a similar way [21]. A meta-analysis and a study of humans revealed that alkali water supplementation may be beneficial for alkalization in people with low urine pH [22]. Another study of volunteers showed that the amount of water drunk may convert to alkaline urine at higher proportions in men compared to women [18]. One limitation of our study is that changes to urine due to increasing or reducing fluid intake amounts, apart from pH, were not studied. In a study, the average urine pH of rats in the control group was 5.79 ± 0.07 [23]. The average urine pH in adult humans can be considered as 6 [24]. Acidity of urine pH may have affected the results of the study. In our study, the second group with tap water pH 7 was used as the control group. Rats are normally fed with this water in the laboratory environment. Since the rats included in the study were of similar weight and age and each group was fed separately in the same cage, a study could be organized in line with their drinking habits, but for this, the rats would have to be kept individually in separate cages. This was not possible due to the physical conditions of the laboratory where we conducted the study. In mammals, whether acidic foods or basic foods are consumed, the pH is kept within very narrow limits to prevent the functions of the cells from being disrupted. Life-threatening blood pH limits are stated as < 6.80 and > 7.70 [25]. Keeping the pH value within this range is defined as homeostasis of the body, and kidneys, bones and lungs play an important role in this. This may be due to the fact that drinking water did not cause significant changes in urine pH value, as in our study. While organizing our study as an experimental study ensures that fluid intake other than water is controlled, it has the disadvantage of not being able to adjust the amount of water. Another limitation of the study is that no parameters other than pH were examined in urine.

## Conclusions

The pH of drinking water does not cause a regular change in urine pH; however, it causes changes to urine pH on different days. Since our study was an experimental study, only drinking water was used as liquid. Drinking water consumed as needed may not have made a permanent change due to hemostasis mechanisms. There is a need to compare experimental study results with clinical studies.
